# Few-shot short utterance speaker verification using meta-learning

**DOI:** 10.7717/peerj-cs.1276

**Published:** 2023-04-21

**Authors:** Weijie Wang, Hong Zhao, Yikun Yang, YouKang Chang, Haojie You

**Affiliations:** 1 School of Computer and Communication, Lanzhou University of Technology, Lanzhou, China; 2 School of Information Science & Engineering, Lanzhou University, Lanzhou, China

**Keywords:** Speaker verification, Meta-learning, Support set, Prototypical network, Global classification, Episodic training strategy

## Abstract

Short utterance speaker verification (SV) in the actual application is the task of accepting or rejecting the identity claim of a speaker based on a few enrollment utterances. Traditional methods have used deep neural networks to extract speaker representations for verification. Recently, several meta-learning approaches have learned a deep distance metric to distinguish speakers within meta-tasks. Among them, a prototypical network learns a metric space that may be used to compute the distance to the prototype center of speakers, in order to classify speaker identity. We use emphasized channel attention, propagation and aggregation in TDNN (ECAPA-TDNN) to implement the necessary function for the prototypical network, which is a nonlinear mapping from the input space to the metric space for either few-shot SV task. In addition, optimizing only for speakers in given meta-tasks cannot be sufficient to learn distinctive speaker features. Thus, we used an episodic training strategy, in which the classes of the support and query sets correspond to the classes of the entire training set, further improving the model performance. The proposed model outperforms comparison models on the VoxCeleb1 dataset and has a wide range of practical applications.

## Introduction

With the widespread application of information technology, there are more and more scenarios that require user identity verification, such as online payments and application logins. In biometric verification methods, speaker verification (SV) (Sarkar & Tan, 2021) technology has incomparable advantages of convenience and non-contact over other verification methods, such as fingerprint recognition. The goal of SV is to verify whether a speaker given test sample is the enrolled speaker given a few utterances for each speaker. However, existing SV methods need to use long speech of more than 15 s or tens of utterances to perform more accurately, which limits the wide application of the SV method. Therefore, researching for short utterances within 10 s, or even short utterances of 2 to 5 s, is of great significance to SV technology (Das & Prasanna, 2018; Poddar, Sahidullah & Saha, 2018; Liu et al., 2022).

Conventional SV methods such as i-vector (Lei et al., 2014; Dehak et al., 2010), Gaussian mixture model (Reynolds, Quatieri & Dunn, 2000) generally adopted a shallow model to directly model probability of the data distribution. At present, most SV methods adopt deep learning, with a few using meta-learning. Researchers have proposed various neural network architectures to extract important speaker information. The widely used architectures include the time delay neural network (TDNN) (Liu et al., 2022; Desplanques, Thienpondt & Demuynck, 2020; Garcia-Romero et al., 2020), ResNet (Chung, Nagrani & Zisserman, 2018; Cai, Chen & Li, 2018; Xie et al., 2019), Transformer (Han, Chen & Qian, 2022; Zhang et al., 2022; Wang et al., 2022; Ranaldi & Pucci, 2023) and long short-term memory (LSTM) networks (Wan et al., 2018). Most of the existing literatures are based on the above network structures improvement or hybrid networks (Bai & Zhang, 2021; Ohi et al., 2021). In addition, researchers have proposed aggregation strategies based on the network architecture that aggregate frame-level features into utterance-level embeddings, such as attention statistical pooling (ASP) (Okabe, Koshinaka & Shinoda, 2018), self-attention pooling (SAP) (Kwon et al., 2021), and temporal average pooling (Chung, Nagrani & Zisserman, 2018), multi-head attention pooling (India, Safari & Hernando, 2021), and multi-resolution multi-head attention pooling (Wang et al., 2020) to represent speaker embeddings. The attention mechanism is used not only in the pooling layers, but also in constructing channel-wise attention module (Thienpondt, Desplanques & Demuynck, 2021), frequency-temporal convolution attention (Yadav & Rai, 2020) or frequency-channel attention module (Liu et al., 2022) to extract fine-grained speaker embeddings. In addition to building neural network architectures, researchers have designed a series of objective functions to help the network to learn features more effectively.

One of the most popular meta-learning methods is prototypical networks (Ko, Chen & Li, 2020), which learns an embedded network that transforms original input into metric space representation. In the metric space, classification is performed by calculating the distance from the prototype center of each class to be tested (the classification loss function in this process is called prototypical network loss). Kumar et al. (2020) have used prototypical network (PN) as a generalized learning method for speaker embedding. Ko, Chen & Li (2020) have used PN for the first time for SV tasks. When the number of samples for each speaker is limited, the performance of PN is better than traditional methods. Kye et al. (2020) used PN and global classification over the whole samples that achieved significant performance for speaker recognition with imbalance length pairs.

The existing short utterance SV methods based on deep learning depend on large-scale datasets with thousands of speakers or tens of thousands of utterances (Xie et al., 2019; Nagrani et al., 2017). In addition, the number of speakers in task is usually large, while the classification objective of deep learning represents a single task, limiting the diversity of training tasks. Unlike deep learning methods, meta-learning aims to enhance the learning algorithm itself by considering the experience of multiple tasks. By training different meta-tasks, meta-learning achieves fast generalization ability (Kumar et al., 2020). However, optimizing only for classes in given meta-tasks may not be sufficient to distinguish speakers. Thus, we perform a process called global classification (GC) in an episodic manner, using the classes of the support set and the query set that correspond to the classes of the entire training set. Kye et al. (2020) used global classification to solve the problem that speaker recognition models perform poorly in real-world scenarios when the length of the enrollment utterance and the test utterance is imbalanced. Their model was trained to match long-short utterance and achieved significant performance gains. We used PN and global classification with episodic training for few-shot short utterance speaker verification (SV). It is worth noting that a good embedding model can adjust the distance between class prototypes, making it easier to classify prototypes. ECAPA-TDNN has good feature extraction capabilities for either SV task with channel and context-dependent attention mechanisms, Squeeze Excitation (SE), multi-layer feature aggregation, and residual blocks. Therefore, it is used to learn meta-task embeddings for few-shot short utterances SV. The distance between a query and its prototype is closer than the distance between the unknown speaker and the prototype in the metric space.

In summary, our main contributions are as follows:

(1) We formulate a meta-learning approach with episodic training for few-shot short utterance SV. Meta-learning considers the experience of many meta-tasks, which helps distinguish speakers.

(2) ECAPA-TDNN is used to implement a nonlinear mapping of the original input to the embedding space on the meta-tasks, making the class prototypes far apart from each other in the embedding space, while each query sample clusters toward the same class prototype group. We call ECAPA-TDNN-inspired Prototypical network as ETP.

(3) An episodic training strategy is designed to optimize the model for generating discriminative speaker features, which combines prototypical network and global classification.

## Preliminary

In this section, we introduce meta-learning, focusing on how it differs from machine learning methods in terms of definition and speaker verification protocol. Meanwhile, metric-based meta-learning is discussed. To make the narrative clearer, the frequently used notations in Section 2 are illustrated in Table 1.

### Meta-learning

Meta-learning is usually understood as “learning to learn”, which aims to learn from the experience of historical tasks, so that the model can learn how to better acquire knowledge and learn new tasks quickly, while ensuring the accuracy of the algorithm (Kumar et al., 2020; Hospedales et al., 2020). In short, learn how to learn across tasks.

To further explain the concept of meta-learning, machine learning and meta-learning are compared. Machine learning learns a model from a dataset *D* = {(*x*_1_, *y*_1_), (*x*_2_, *y*_2_), …, (*x*_*N*′_, *y*_*N*′_)}. Given inputs and labels, a predictive model $\hat {y}={f}_{\theta }(x)$ with hyperparameters *θ* is trained, in order to get the predicted values as close to the true value as possible. The optimal model parameters are as follows:

(1) \begin{eqnarray*}{\theta }^{\ast }=\arg \nolimits ~{\min \nolimits }_{\theta }~L(D;\theta ,\phi )\end{eqnarray*}

where *L* (•) is the loss function that computes the error of the true and predicted values, and *ϕ* is pre-specified.

**Table 1:** The mathematical notions and parameters used in the Section 2 are summarized.

Notations	Description
*D*, *D*_*t*_	The whole dataset for machine learning, the *t*-th meta-task (or episode) dataset.
*S, Q*	The support set *S*, the query set *Q*.
*x, y*	Sample, label.
*f*_*θ*_ ( ⋅ )	Predictive model with hyperparameters *θ*.
*F*_*ϕ*_( ⋅ )	The learning algorithm *F*_*ϕ*_( ⋅ ) that can learn the base model, *ϕ* is learnable hyperparameters.
*L* ( ⋅ )	Loss function.
*d*_*θ*_ ( ⋅ )	Metric function.

**Figure 1:**
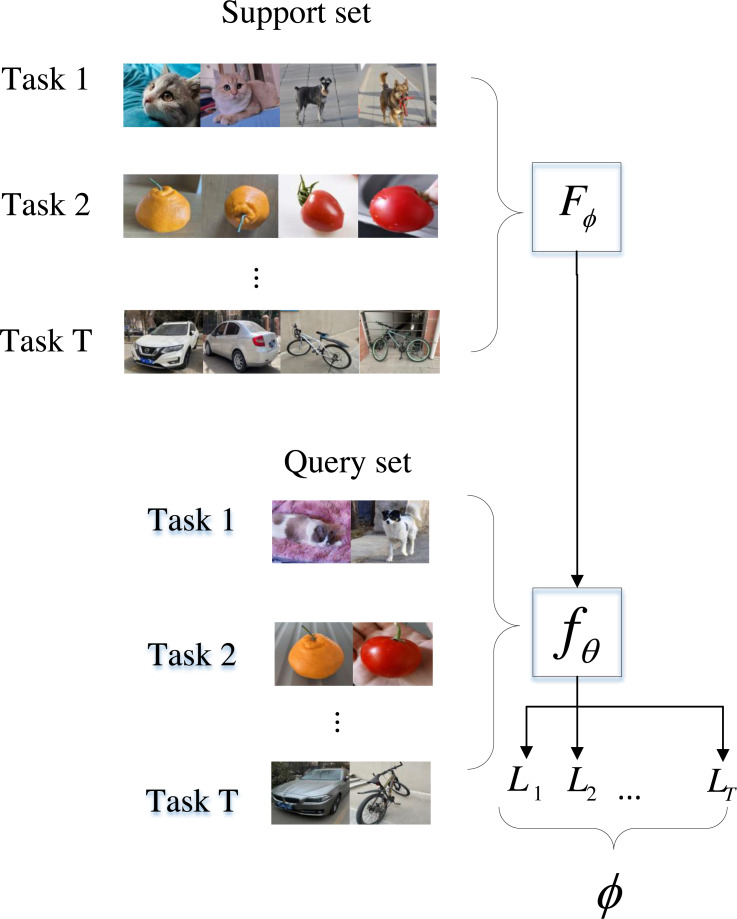
Meta-train phase.

Meta-learning transfers knowledge across tasks, rather than learning from scratch for each task (Baik et al., 2021). It is assumed that *ϕ* is learnable rather than pre-specified. Figure 1 shows the meta-train phase. Image is more intuitive than speech, so image classification is used as an example. Given T meta-tasks (or called episodes) denoted as $\{ {D}_{t}\} _{t=1}^{T}$, researchers train a learning algorithm *F*_*ϕ*_(•) that can learn the base model $\hat {y}={f}_{{\theta }^{\ast }}(x)$, by solving:

(2) \begin{eqnarray*}{\phi }^{\ast }=\arg \nolimits ~{\max \nolimits }_{\phi }~\log \nolimits ~p(\phi {|}\{ {D}_{t}\} _{t=1}^{T}).\end{eqnarray*}

Each meta-task (or episode) dataset is denoted as *D*_*t*_ = (*S, Q*)^(t)^, consisting of a training set and a test set, also known as the support set *S* and the query set *Q*. The support set is used for learning and training *F*_*ϕ*_(•). The query set is used to calculate the loss of model *f*_*θ*_ (⋅ ) learned by *F*_*ϕ*_(•). According to the loss value, the model parameters are updated by backpropagation. The *t*-th meta-task base model parameters are as follows:

(3) \begin{eqnarray*}{{\theta }^{\ast }}^{ \left( t \right) }=\arg \nolimits ~{\max \nolimits }_{\theta }{\sum \nolimits }_{(x,y)\in {Q}^{ \left( t \right) }}\log \nolimits ~p(y{|}{\phi }^{\ast },x).\end{eqnarray*}

In summary, in the base learning process, base tasks such as speaker recognition defined by a single task dataset and training objectives are solved. In the meta-learning process, the meta-task based on the meta-objective and meta-task datasets is to update the base model (Sun et al., 2019; Lang et al., 2022). Most meta-learning methods are applied to few-shot tasks (Chang et al., 2022). The model trained by a small number of samples can quickly adapt and master the new few-shot task. The architecture of the meta-learning model is similar to the deep learning model. It is logically divided into classifier and feature extractor. The feature extractor is composed of a deep neural network.

### Metric-based meta-learning

Metric-based meta-learning aims to learn an embedding network that transforms the raw input into a metric space representation. In the metric space, the class is predicted by comparing the similarity between query set samples and support set samples. The most popular metric-based meta-learning methods include prototypical networks, siamese networks (Koch, Zemel & Salakhutdinov, 2015), relation networks (Sung et al., 2018), and matching networks (Vinyals et al., 2016).

The predicted probability over a set of known labels *y* is a weighted sum of labels of support set samples. The weight is generated by the metric function *d*_*θ*_ (⋅ ) that computes the similarity between two samples.

(4) \begin{eqnarray*}{P}_{\theta }(y{|}x,S)={\sum }_{({x}_{i},{y}_{i})\in S}{d}_{\theta }(x,{x}_{i}){y}_{i}.\end{eqnarray*}

### Speaker verification protocol

The SV based on deep learning process can be divided into three phases: During the training phase, a large number of speaker utterances are fed into the neural network, which learns a predictive model to classify the speakers. During the enrollment phase, the new speaker (different from the speaker in the training phase) utterances are inputted into the trained model without the classification for generating a new speaker model. Each new speaker has its speaker model. During the evaluation phase, the utterance to be verified is inputted into the trained model to obtain its embedding representation. Then, we calculate the similarity between the embedding of the utterance to be tested and the target speaker model, judging whether the speaker is the target speaker according to the similarity score and the preset threshold. If the score exceeds the threshold, it is confirmed that the speaker of the utterance being tested is the target speaker, and vice versa.

The SV process based on meta-learning is different from the SV based on deep learning, including meta-train SV phase and meta-test SV phase. During the meta-train phase, a large number of training meta-task sets are inputted into the neural network. In each episode, the support set is used for training model *F*_*ϕ*_(•). The query set is used for calculating the loss of model *f*_*θ*_ (⋅ ) learned by the learning algorithm *F*_*ϕ*_(•). The loss values of all meta-tasks are added to obtain the model loss value. According to the loss, the model parameters are updated by backpropagation until convergence, and thus the model is successfully trained. During the meta-test phase, in each episode, the support set is used for adapting the new SV meta-learner. The query set is used for evaluating the performance of the meta-learner for fast adaptation to unseen SV tasks.

## Method

### Problem setup

Suppose that *D* is the entire training set, which is divided into several episodes to mimic few-shot SV task. In each episode, *N* speakers are randomly selected from the training set. *K+M* samples are randomly selected for each speaker. Meta-tasks include support set *S* = {*S*_1_, …, *S*_*N*_} and query set *Q* = {*Q*_1_, …, *Q*_*N*_}. ${S}_{\mathrm{n}}=\{ ({x}_{1}^{s},{y}_{n}^{s}),\ldots ,({x}_{K}^{s},{y}_{n}^{s})\} $, ${Q}_{n}=\{ ({x}_{1}^{q},{y}_{n}^{q}),\ldots ,({x}_{M}^{q},{y}_{n}^{q})\} $ respectively represent the labeled sample set of the *n*-th speaker in the support set and the query set. *K*, *M* is respectively the number of utterances of *S*_*n*_, *Q*_*n*_.*x*_*n*,*i*_ represents the *i*-th utterance of the *n*-th speaker. *y* is the corresponding label of *x*_*n*,*i*_, *y*_*n*_ = *n*.

### Learning embedding for few-shot short utterances SV

**Figure 2:**
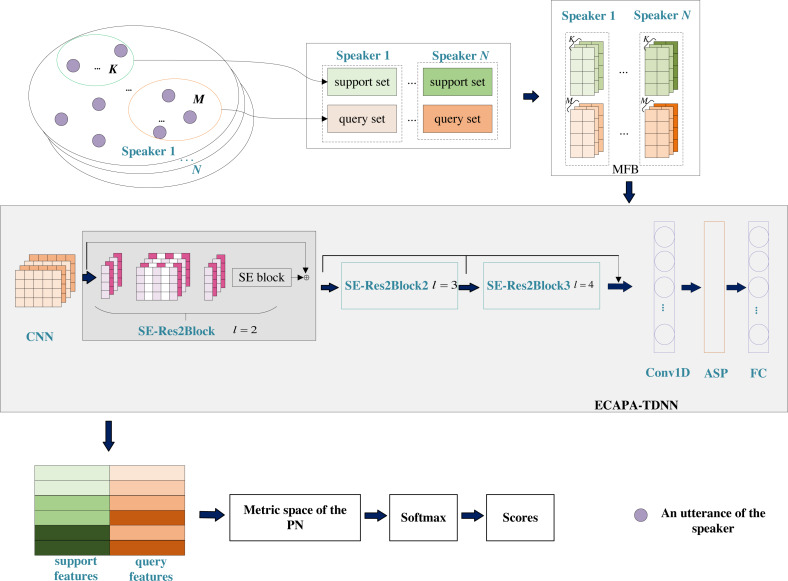
Network architecture of ETP.

The key to the metric-based meta-learning approach for few-shot SV task is to learn meta-task embeddings (Ye et al., 2020), where embeddings from the same speaker are closer than embeddings from different speakers. Therefore, we learn meta-task embeddings to modify the prototypes to make them easier to distinguish. The overall architecture of ETP is shown in Fig. 2. The raw utterances in the support set and query set are pre-processed (pre-emphasis, frame addition, short-time Fourier transform and Mel-filterbank filtering operations are performed sequentially.) to obtain the Mel-filterbank (MFB) features (Ohi et al., 2021). One utterance corresponds to one MFB feature matrix with 80 rows and *H* columns. 80 is the dimension of a frame of MFB features, and *H* is the number of frames. MFB feature matrix is used as the input of ETP for feature extraction. We propose ETP, which integrates ECAPA-TDNN into the prototypical network to implement a nonlinear mapping of the original input to the metric space on the meta-tasks. The distance between a query and its prototype is closer than the distance between the unknown speaker and the prototype in the metric space.

 ECAPA-TDNN contains the advantages of x-vector and ResNet architecture, adding residual connections between frame-level layers to enhance speaker characteristics and avoid gradient degradation. The convolution kernel of CNN has a fixed height, which is the same as the dimension of the speech frame, to perform convolution along the direction of the frame. We built three SE-Res2Blocks, using one-dimensional dilated convolution with the dilated factors of 2, 3, and 4. The outputs of the three SE-Res2Blocks are connected. The ASP is to introduce an attention mechanism in the statistical pooling layer to calculate the importance of each frame. Then, the attention pooling layer is combined with the standard deviation for aggregation, which can represent the features of any distance in the context to capture the long-term characteristics of speakers more effectively. The output features of ASP are mapped to 256-dimensional features through a fully connected layer (FC).

**Figure 3:**
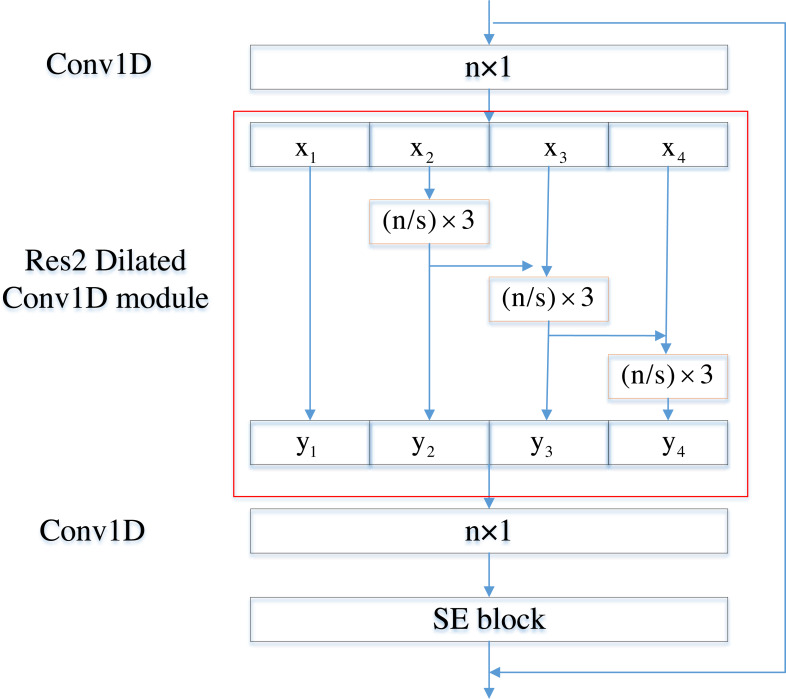
SE-Res2Block architecture.

SE-Res2Block consists of two convolutional layers, Res2 Dilated Conv1D module and SE, which are used to effectively learn feature information. As shown in Fig. 3, the size of the convolution kernel of the two convolution layers is set to c ×1, and the size of the convolution kernel of Res2 Dilated Conv1D is set to (c/s) ×3. Dilated convolution layers with different dilated factors in Res2 Dilated Conv1D can effectively expand the receptive field of the convolution layer without additional computation complexity. We use batch normalization BN and activation function ReLU between layers. In addition, to avoid the gradient vanishing or exploding, a residual connection is constructed.

Res2Dilated Conv1D with the scale dimension processes multi-scale features by hierarchical residual connections internally, which is beneficial to extract local and global information (Gao et al., 2019). It also uses one-dimensional dilated convolution to expand the receptive field and obtain more useful information without changing the size of the convolution kernel (Zhang, Wang & Jung, 2018). The definition of time dilated convolution can be written as:

(5) \begin{eqnarray*}(X{\ast }_{l}w)(t)={\sum }_{p+lq=t}X(p)w(q)\end{eqnarray*}

where *X* represents the speech signal, *w* represents the convolution kernel. *l* represents the dilated convolution factor, which is the interval when the convolution kernel processes the data, *l* ∈ ℤ^+^.

In the Res2 Dilated Conv1D module, the number of the frame x is *H*, x_*i*_ ∈ ℝ^c′×*H*^. We divide x into s subsets x_*i*_, where *i* ∈ {1, 2, …, s}, which replace c-channel convolution kernels with a set of c′-channel convolution kernels (c = s × c′). It changes the number of channels. The convolution kernel group is connected layer by layer. This process is expressed in mathematical form, that is, except for x_1_, each feature subset x_*i*_ has its corresponding convolution kernel *w*_*i*_. We add the current feature subset x_*i*_ and the output result of the previous convolution operation *w*_*i*−1_x_*i*−1_, and then perform the convolution operation with the current convolution kernel. The output after convolution is y_*i*_, until all the feature data is processed. y_*i*_ is shown in formula (6):

(6) \begin{eqnarray*}{\mathrm{y}}_{i}= \left\{ \begin{array}{@{}ll@{}} \displaystyle {\mathrm{x}}_{i}&\displaystyle i=1\\ \displaystyle {w}_{i}{\mathrm{x}}_{i}&\displaystyle i=2.\\ \displaystyle {w}_{i}({\mathrm{x}}_{i}+{\mathrm{y}}_{i-1})&\displaystyle 2\lt i\leq s \end{array} \right. \end{eqnarray*}

All the features y_*i*_ are spliced and sent to a set of convolutional layers with the convolution kernel of c ×1 for information fusion to obtain feature data. Since the convolutional layer does not effectively use the channel information of the features, SE is introduced to obtain the channel relationship and improve the performance of the task system. First, global average pooling is used to compress global spatial information to channel-level statistical information (Hu, Shen & Sun, 2018). The squeeze operation reduces the time dimension to generate statistics *z* ∈ ℝ^*C*^. The *c*-th channel of z is given by:

(7) \begin{eqnarray*}{z}_{c}={\mathrm{F}}_{sq}({u}_{c})= \frac{1}{T} {\mathop{\sum \nolimits }\nolimits }_{t=1}^{T}{u}_{c,t}\end{eqnarray*}

where *u*_*c*_ represents the *c*-th channel characteristic of *U*.

Secondly, two FCs are used to capture the interdependencies between the channels and assign corresponding weights to each channel feature. This process is an excitation operation as follows:

(8) \begin{eqnarray*}s={\mathrm{F}}_{ex}(z,W)=\sigma ({W}_{2}\delta ({W}_{1}z+{b}_{1})+{b}_{2})\end{eqnarray*}

where *σ* is the sigmoid function, *δ* is the ReLU function, and *F*_*ex*_ (⋅ ) represents an excitation operation.

Finally, the weight information of each feature channel is multiplied by the feature information, so that the network can selectively focus on important features and suppress unnecessary features, to achieve adaptive calibration of feature channels. The multiplication of feature *u*_*c*_ and scalar *s*_*c*_ is shown in formula (9):

(9) \begin{eqnarray*}\widetilde {{u}_{c}}={s}_{c}\cdot {u}_{c}.\end{eqnarray*}

### Episodic training

**Figure 4:**
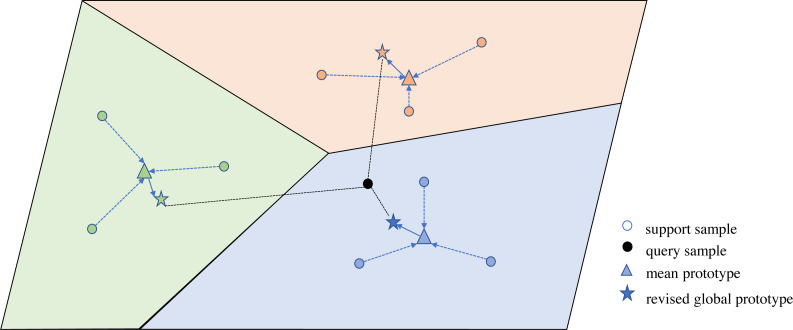
Metric space of the prototypical network.

We use prototypical network to be trained in an episodic manner. Metric space of the prototypical network is shown in Fig. 4. The prototypical network learns a metric space, calculating the distance from the prototype center of each speaker to be tested speech.

Firstly, the prototype center *p*_*n*_ of the speaker is calculated, which is the average of all samples in each type of support set, as shown in formula (10):

(10) \begin{eqnarray*}{p}_{n}= \frac{1}{K} {\sum }_{{x}_{n,i}\in {S}_{n}}f({x}_{n,i}^{s})\end{eqnarray*}

where *n* = 1, 2, …, *N*, *i* = 1, 2, .., *K*, *f*(⋅) is the model required for SV, which is inputted MFB features to extract speaker features. Then the distance distribution between each query sample and the prototype center of the *N* speaker is calculated as shown in formula (11):

(11) \begin{eqnarray*}p({y}_{n}^{q}=n{|}{x}_{n,i}^{q})= \frac{\exp \nolimits (d(f({x}_{n,i}^{q}),{p}_{n}))}{{\mathop{\sum \nolimits }\nolimits }_{{n}^{{^{\prime}}}}^{N}\exp \nolimits (d(f({x}_{n,i}^{q}),{p}_{{n}^{{}^{{^{\prime}}}}}))} \end{eqnarray*}

where, *d* (⋅ ) is a cosine distance measurement function for measuring between the query sample and the center of the class prototypes. Finally, the loss of calculating the sub-task is:

(12) \begin{eqnarray*}{J}_{meta}= \frac{1}{N} {\mathop{\sum }\nolimits }_{n=1}^{N} \frac{1}{M} {\sum }_{({x}_{n,i}^{q},{y}_{n}^{q})\in {Q}_{n}}-\log \nolimits ~p({y}_{n}^{q}{|}{x}_{n,i}^{q}).\end{eqnarray*}

Given a support set containing the target class, we calculate the prototype center of each target class and classify it according to the closest metric distance. However, only optimizing the meta-task model cannot be sufficient to distinguish speakers. Therefore, it is necessary to globally classify each sample of each meta-task against the whole dataset, so that the model can better recognize the speaker. Assume that each class has a set of global prototypes *ω* = {w_*n*_ ∈ *R*^*d*^|*n* = 1, …, *N*′}, *N*′ is the number of speakers in the entire training set. *d* is the dimension of the speaker feature. Then the probability that the utterance *x* is the class *y*:

(13) \begin{eqnarray*}p(y{|}x;\omega )= \frac{\exp \nolimits (d(f(x),{\mathrm{w}}_{y}))}{{\mathop{\sum \nolimits }\nolimits }_{{n}^{{}^{{^{\prime}}}}=1}^{{N}^{{}^{{^{\prime}}}}}\exp \nolimits (d(f(x),{\mathrm{w}}_{{n}^{{}^{{^{\prime}}}}}))} .\end{eqnarray*}

Then, the global loss is calculated as Eq. (14):

(14) \begin{eqnarray*}{J}_{global}(\omega )= \frac{1}{K+M} {\sum }_{(x,y)\in S\cup Q}-\log \nolimits p(y{|}x;\omega ).\end{eqnarray*}

Finally, the loss of the meta-task and the global loss are added as follows:

(15) \begin{eqnarray*}J={J}_{meta}+{J}_{global}(\omega ).\end{eqnarray*}

## Experiments

### Datasets

The VoxCeleb2 and VoxCeleb1 datasets (Nagrani et al., 2017), which have no identical speakers between them, are used for the experiments. VoxCeleb2 was published by the University of Oxford in 2018 and contains 1,128,246 utterances from 5,994 speakers downloaded from YouTube. VoxCeleb1 contains 153,516 utterances for 1,251 speakers, which is composed of VoxCeleb1 test set and dev set. Speech is highly variable and contains various background noises. The average length of the full utterances of VoxCeleb1 and VoxCeleb2 are 8.2 and 7.8, respectively. SITW (McLaren et al., 2016) contains 299 speakers with average of 8 voices each. The speech is collected in complex scenes with noise, reverberation, *etc.*

### Data representation

This article uses 80-dimensional MFB features with a 25 ms window and a 15 ms frame shift as the input of the model. We normalized the speech frame by subtracting the average value and dividing it by the standard deviation of all frequency components, without performing any voice activity detection (VAD) operation and data augmentation. During the training process, we set 1-shot 100-way in each episode and the number of query samples to 2. Set the length of the utterance to 2 s. If the duration of the utterance is less than 2 s, this utterance segment is copied to a duration of 2 s.

### Implementation details

We implement a model with 512 channels in the convolutional layers using PyTorch. When only the global classification objective is used, the mini-batch size is 256. When combining PNL and GC optimize model, the episode size is 100. We use the SGD optimizer with the momentum set to 0.9 and use the weight decay set to 2e−4. Set the initial value of the learning rate to 0.1 and its decay rate to 10 until convergence. The experiment was done with NVIDIA V100 and T4 GPU.

### Baseline models

**x-vector.** The pre-trained x-vector model except for the final layer is used as an initialization model (Kumar et al., 2020). The Adam optimizer with an initial learning rate of 1e−3. The learning rate is reduced to 1e−6. Dropout and batch normalization are used at all layers for regularization.

**ThinResNet-34.** The model is trained using the Adam optimizer. The initial learning rate of 0.001 is reduced by 10 after every 36 epochs until convergence. The mini-batch size is 160.

### Evaluation metrics

Equal error rates (EER) and detection cost function (DCF) are applied to evaluate the performance of speaker verification systems (Xu et al., 2021). The evaluation metrics EER and DCF refer to two parameters, which are False Acceptation Rate (FAR) and False Rejection Rate (FFR). FAR is the percentage of acceptance in the sample that should not be accepted. FRR is the percentage of rejection in the sample that should not be rejected. The EER is equal to the value when FAR and FRR are equal (Avila, O’Shaughnessy & Falk, 2021). The lower the EER value, the better the performance of the system is required.

## Result

### The impact of feature dimensions

The ETP is trained on the VoxCeleb1 dataset and tested on the original test set of VoxCeleb1 which contains 37,720 full utterances from 40 speakers. To evaluate the impact of feature dimensionality on the SV task, we select and compare 40-dimensional MFB features and 80-dimensional MFB features.

The experimental results in Table 2 show that the performance of the model trained with 80-dimensional MFB performs slightly better than that trained with 40-dimensional MFB features, regardless of which episodic training strategy PNL or GC or combining PNL and GC is used to optimize the model. It indicates the effectiveness of increasing data dimension. Data with larger data dimensions contain more speaker information, taking up more disk space and requiring more computation, but the model performance is not significantly improved, which may represent that data with larger dimensions are sparser than data with smaller dimensions.

### Verification on VoxCeleb1

The model is trained on the VoxCeleb2 dataset and evaluated on three different test lists from the VoxCeleb1 data set and eval core-core trial pairs of SITW dataset: (1) the original test list; (2) the expanded VoxCeleb1-E list contained training sets and VoxCeleb1 test set; and (3) the challenging VoxCeleb1-H list. In addition, there are a few errors in the VoxCeleb1-E and VoxCeleb1-H lists. Xie et al. cleaned up the errors and publicly released the cleaned test lists. We do not add any speech time, which may result in performance improvement.

Table 3 shows the performance of models on the original test set of VoxCeleb1. We use short utterance training our models to evaluate the performance of the model on full utterances. ETP exceeds the ThinResNet-34 (Xie et al., 2019) and ResNet-50 (Chung, Nagrani & Zisserman, 2018) models (EER is 2.36% *vs* 3.22% and 4.19%). ETP and x-vector are both meta-learning methods. ETP with episodic training strategy PNL is comparable to the x-vector (EER is 3.46% *vs* 3.48%). When combining PNL and GC jointly to optimize the model, ETP outperforms the x-vector (EER is 2.36% *vs* 3.48%), indicating the effectiveness of GC. GC enhances information transfer across meta-tasks by each sample of each meta-task against the whole dataset, improving the performance of the model. Similarly, the last two rows of Table 3 show that the combination of PNL and GC to train the model outperforms the single PNL.

**Table 2:** Performance comparison on effects of data dimensions.

Data dimensions	P		G		P+G	
	EER%	DCF	EER%	DCF	EER%	DCF
40D	2.74	0.316	2.67	0.312	1.37	0.180
80D	2.56	0.294	2.48	0.279	1.20	0.146

**Notes.**

D Dimension P Prototypical Network Loss G Global classification G +P Global classification and Prototypical Network Loss EER Equal Error Rates DCF Detection Cost Function

**Table 3:** Results for verification on the full utterance.

Architecture	Loss	Aggregation	Dims	EER%	DCF
ThinResNet-34 (Xie et al., 2019)	Softmax	GhostVLAD	512	3.22	–
ThinResNet-34 (Xie et al., 2019)	Softmax	TAP	512	10.48	–
ResNet-34 (Chung, Nagrani & Zisserman, 2018)	Softmax+C	TAP	512	5.04	–
ResNet-50 (Chung, Nagrani & Zisserman, 2018)	Softmax+C	TAP	512	4.19	0.449
x-vector (Kumar et al., 2020)	P	SP	512	3.48	0.331
ETP	P	ASP	256	3.46	0.359
ETP	G+P	ASP	256	**2.36**	**0.241**

**Notes.** Bold values represent the optimal values, which are highlighted for emphasis.

P Prototypical Network Loss G+P Global classification and Prototypical Network Loss C Contrastive loss AAM Additive Angular Margin ASP Attentive Statistics Pooling TAP Temporal Average

Table 4 shows the comparison results of model performance on VoxCeleb-E, VoxCeleb-H test sets, the cleaned test sets and SITW eval dataset. VoxCeleb1-E contains a large number of expanded utterances, which can be used to fully test the performance of models. It is difficult to evaluate the model on the VoxCeleb1-H list, due to it contains speakers from the same gender and nationality, which the similarity between speakers is high. ETP outperforms ThinResNet-34 and ResNet-50 in all cases. ETP can be generalized for target tasks and further enhance performance during the testing phase of SV.

**Table 4:** Results for verification on VoxCeleb-E, VoxCeleb-H and SITW.

Architecture	Loss	VoxCeleb-E		VoxCeleb-E*		VoxCeleb-H		VoxCeleb-H*		SITW	
		EER%	DCF	EER%	DCF	EER%	DCF	EER%	DCF	EER%	DCF
ThinResNet-34 (Xie et al., 2019)	Softmax	3.25	–	3.24	–	5.17	–	5.06	–	4.98	0.539
ResNet-50 (Chung, Nagrani & Zisserman, 2018)	Softmax+C	4.43	0.524	–	–	7.43	0.673	–	–	6.78	0.667
ETP	G+P	**2.41**	**0.276**	**2.27**	**0.262**	**4.15**	**0.372**	**4.03**	**0.356**	**3.90**	**0.428**

**Notes.** Bold values represent the optimal values, which are highlighted for emphasis.

*cleaned up versions of the test lists by Xie et al. (2019).

### Verification based on the length of short utterances

We randomly sample 100 positive sample pairs and 100 negative sample pairs in the VoxCeleb1 dataset to obtain test sample pairs, testing the performance of models. Randomly cut the test speech for 1 s, 2 s, and 5 s. If the length of the test utterance is shorter than the required length, copy the utterance segment itself and set it as the target length.

Table 5 shows the effect of the length of short utterances on the performance. ETP outperforms baseline models in all cases. The episodic training manner is helpful for mining the novel speaker information from few-shot SV tasks to improve the discriminative ability of prototypes. Meanwhile, when the utterance length is 5 s, all models achieve the lowest EER value. There is a strong correlation between model performance and utterance length. With the increase of utterance length, more relevant speech signals from speaker are captured so that the EER value is lower. To prove the effectiveness of the episodic training strategy of PNL and GC, the ablation experiment is implemented. The experiment on 1 s, 2 s, and 5 s utterances or full utterances (Table 3) shows that the method with the prototypical network and global classification is more effective than the method using the prototypical network or global classification alone. The episodic training manner can make the distance between a query and its prototype closer than between the unknown speaker and the prototype in the metric space, effectively distinguishing speakers.

**Table 5:** Length of short utterances on performance.

Architecture	Loss	Aggregation	1s	2s	5s
			EER%	EER%	EER%
ThinResNet-34 (Xie et al., 2019)	Softmax	GhostVLAD	12.72	6.59	3.36
x-vector (Kumar et al., 2020)	P	SP	8.35	5.41	3.97
ETP	P	ASP	8.19	5.30	3.92
ETP	G	ASP	7.73	4.95	3.71
ETP	G+P	ASP	**6.44**	**4.16**	**3.34**

**Notes.** Bold values represent the optimal values, which are highlighted for emphasis.

### Ablation experiment

In order to measure the effectiveness of the Res2 Dilated Conv1D module in the few-shot short utterances speaker verification task, ablation experiments are performed on the models. The Res2Dilated Conv1D module is replaced by a common one-dimensional convolutional layer.

As shown in Table 6, the Res2 Dilated Conv1D module significantly improves the performance of the model when it is testing the full utterances in the three different datasets of VoxCeleb1. The results in the second column of Table 7 show that the parameters of the ETP are reduced by 23.2%. The results in the fifth column of Table 7 show that when the prototypical network loss is combined with the global classification, the EER of ETP is relatively reduced by 14.5% than that of the NR-ETP; when using only the PNL, the performance of the ETP is relatively 0.96% higher than that of the model the NR-ETP. It is proved that the multi-scale features extracted by Res2 Dilated Conv1D represent the personality information of the speaker, which improves the performance of the model. ETP and NR-ETP take around 4 days to train.

**Table 6:** Ablation study of ETP on VoxCeleb1. NR-ETP: EPT without Res2Dilated Conv1D.

Architecture	Loss	VoxCeleb1		VoxCeleb-E		E*		VoxCeleb-H	
		EER%	DCF	EER%	DCF	EER%	DCF	EER%	DCF
NR-ETP	G+P	2.98	0.339	2.93	0.325	2.84	0.324	4.87	0.431
ETP	G+P	**2.36**	**0.241**	**2.41**	**0.276**	**2.27**	**0.262**	**4.15**	**0.372**

**Notes.** Bold values represent the optimal values, which are highlighted for emphasis.

**Table 7:** Ablation study of ETP on short utterances. NR-ETP: EPT without Res2Dilated Conv1D.

Architecture	Params	Loss	Test	1s	2s	5s
				EER%	EER%	EER%
NR-ETP	6.9M	P	Vox1	8.27	5.57	3.98
NR-ETP	6.9M	G+P	Vox1	7.59	5.13	3.85
ETP	5.3M	P	Vox1	8.19	5.30	3.92
ETP	5.3M	G+P	Vox1	**6.44**	**4.16**	**3.34**

**Notes.** Bold values represent the optimal values, which are highlighted for emphasis.

Vox1 VoxCeleb1 dev sets and VoxCeleb1 test sets

## Conclusion

In this article, we used the meta-learning method for solving the few-shot short utterances SV task. We sampled from the training set to construct a large number of new subtasks to mimic few-shot scenario. ECAPA-TDNN was applied to the prototypical network to learn meta-task embeddings for either meta-task, where embeddings from the same speaker are closer than embeddings from different speakers. We used global classification and prototypical network in an episodic manner to train a model to obtain discriminative speaker features. The SV task was tested on the VoxCeleb1 dataset. The experimental results show that the performance of this model is better than the comparison model.

##  Supplemental Information

10.7717/peerj-cs.1276/supp-1 Supplemental Information 1 Datasets

10.7717/peerj-cs.1276/supp-2 Supplemental Information 2 Codes for data pre-processing, feature extraction and model

## Additional Information and Declarations

### Data Availability

The following information was supplied regarding data availability: The VoxCeleb2 and VoxCeleb1 datasets are available at https://www.robots.ox.ac.uk/ vgg/data/voxceleb/. The SITW dataset is available at http://www.speech.sri.com/projects/sitw/.
