# Supplementary material for: Few-shot short utterance speaker verification using meta-learning
Source: PeerJ Comput Sci. 2023 Apr 21;9:e1276. doi: 10.7717/peerj-cs.1276 (PMC10280689; doi:10.7717/peerj-cs.1276)
Supplement: Supplemental Information 1 [file peerj-cs-09-1276-s001.docx]

The VoxCeleb2, VoxCeleb1 and SITW datasets are used for the experiments, which are public datasets. The VoxCeleb2 and VoxCeleb1 datasets are available at <https://www.robots.ox.ac.uk/~vgg/data/voxceleb/>. The SITW dataset is available at http://www.speech.sri.com/projects/sitw/.
